# Opportunities, Enablers, and Barriers to the Use of Recorded Recovery Narratives in Clinical Settings

**DOI:** 10.3389/fpsyt.2020.589731

**Published:** 2020-10-30

**Authors:** James Roe, Susan Brown, Caroline Yeo, Stefan Rennick-Egglestone, Julie Repper, Fiona Ng, Joy Llewelyn-Beardsley, Ada Hui, Pim Cuijpers, Graham Thornicroft, David Manley, Kristian Pollock, Mike Slade

**Affiliations:** ^1^National Institute for Health Research, Applied Research Collaboration (ARC) East Midlands, University of Nottingham, Nottingham, United Kingdom; ^2^Mindtech MedTech Cooperative, Institute of Mental Health, University of Nottingham, Nottingham, United Kingdom; ^3^School of Health Sciences, Institute of Mental Health, University of Nottingham, Nottingham, United Kingdom; ^4^Implementing Recovery Through Organisational Change (ImROC), Nottingham, United Kingdom; ^5^Department of Clinical, Neuro and Developmental Psychology, Amsterdam Public Health Research Institute, Vrije Universiteit Amsterdam, Amsterdam, Netherlands; ^6^Centre for Global Mental Health and Centre for Implementation Science, Health Service and Population Research Department, Institute of Psychiatry, Psychology and Neuroscience, King's College London, London, United Kingdom; ^7^Nottinghamshire Healthcare NHS Foundation Trust, Nottingham, United Kingdom; ^8^School of Health Sciences, University of Nottingham, Nottingham, United Kingdom

**Keywords:** education and training, clinical practice, mental health, online resources, recovery narrative

## Abstract

**Background:** Recorded Recovery Narratives (RRNs) describing first-person lived experience accounts of recovery from mental health problems are becoming more available. Little is known about how RRNs can be used in clinical practice and clinical education.

**Aims:** The aim of this paper is to enable implementation planning for RRN interventions by identifying determinants of uptake. The objective was to identify opportunities, barriers, and enablers to the uptake of RRN interventions in clinical practice and education.

**Method:** Three phases of focus groups were conducted with multi-professional mental health clinicians. Phase 1 (4 groups, *n* = 25) investigated current and possible uses of RRNs, Phase 2 (2 groups, *n* = 15) investigated a specific intervention delivering recovery narratives. Phase 3 (2 groups, *n* = 12) investigated clinical education uses. Thematic analysis was conducted.

**Results:** RRNs can reinforce the effectiveness of existing clinical practices, by reducing communication barriers and normalizing mental health problems. They can also extend clinical practice (increase hope and connection, help when stuck). Clinical considerations are the relationship with care pathways, choice of staff and stage of recovery. In educational use there were opportunities to access lived experience perspectives, train non-clinical staff and facilitate attitudinal change. Barriers and enablers related to design (ability to use online resources, accessibility of language, ability to individualize choice of narrative), risk (triggering content, staff skills to respond to negative effects), trust in online resource (evidence base, maintenance), and technology (cost of use, technology requirements).

**Conclusions:** RRNs can both improve and extend existing clinical practice and be an important educational resource. RRNs can improve engagement and hope, and address internalized stigma. Beneficially incorporating RRNs into clinical practice and education may require new staff skills and improved technological resources in healthcare settings. Future work could focus on the use of peer support workers views on RRN use and how to avoid unnecessary and unhelpful distress.

**Trial Registration Number:** Work in this paper has informed three clinical trials: ISRCTN11152837; ISRCTN63197153; ISRCTN76355273.

## Introduction

Mental health recovery narratives have been defined as first-person lived experience accounts of recovery from mental health problems, which refer to events or actions over a period of time, and which include elements of adversity or struggle, and of self-defined strengths, successes or survival (1, 2). Recorded Recovery Narratives (RRNs) are those presented in invariant form, such as in prose, poetry, or video (3).

Whilst the focus in the remainder of this paper is on clinical perspectives on RRNs, it is fully acknowledged that the concept of recovery emerged from the survivor movement. One goal of this movement has been the strengthening and emancipation of individuals who have been traumatized by their experience of mental health services, including through telling their individual and collective stories. Recovery has been taken up as a guiding value in mental health systems internationally (4). This implementation of recovery in services has been criticized for several reasons, including that it is a professional co-optation which occludes issues of social justice (5), that it commodifies experiences in systems that sustain subjugation (6), and that it is a cover for neoliberalism (7). These issues have led some groups, such as the critical theorist and activist collective *Recovery in the Bin*, to call for the replacement of recovery with “unrecovery” (8). Whilst recognizing this critique of recovery, the current study concerns the under-investigated views and experiences of clinicians in relation to the use of RRNs, because the attitudes and behavior of clinicians can influence access by service users to RRNs.

A growing number of RRNs are publicly available, both individually and in curated collections (3). One example is the Narrative Story Bank of text-based recovery narratives (9), which was produced by the Scottish Recovery Network for purposes including “inspiring hope,” and “offering tools and techniques for recovery” (10), drawing on knowledge developed through an earlier project by the New Zealand Mental Health Commission (11). These and other RRNs have been described as an important technology in the implementation of recovery-oriented mental health practices in Scotland, where they facilitated a nationally relevant conceptualization to emerge. They also enabled experimentation within clinical services; examples include the integration of recovery narrative writing as a reflexive tool into psychiatric practice, and the production of books of recovery narratives by groups of users of a particular service (10).

A large qualitative study has identified that RRNs can make a positive impact on a recipient by providing access to narrators not readily accessible in everyday life (12), and a systematic review has concluded that clinical use of RRN collections might benefit those who have limited access to peers, either through social or geographical isolation (13). Collections of RRNs are starting to be used in online mental health interventions where they might enable large-scale usage by services users, for example in a website that provided people with experience of psychosis with self-guided access to a range of lived experience videos alongside peer support (14). These intervention might be seen as part of a longer tradition of bibliotherapy, in which reading material such as self-help books (15) and autobiographies (16) is introduced to psychotherapeutic processes. Meta-analyses have shown that bibliotherapy is effective for various clinical populations, including people with unipolar depression (17), alcohol problems (18), and sexual dysfunction (19). Receiving RRNs can reduce various forms of stigma, including self-stigma (12), important given that stigma can lead to a range of negative consequences for people living with mental health problems (20). Recovery narratives, in the form of server user testimonials, have been identified as one of the active ingredients of anti-stigma campaigns (21) which have been shown to be effective (22). RRNs have been introduced to clinical training where they can enhance communication skills and empathy (23). They might therefore be seen as a useful tool for aligning clinical education with a recovery orientation (24).

Despite some of the possibilities and practical examples illustrated above, and despite widespread use in anti-stigma campaigns, there is no evidence to suggests that RRNs are considered to be a routine part of clinical practice or clinical training. Whilst this could of course reflect a carefully-considered clinical position, it may also be that a useful intervention has not successfully moved from development into practice due to factors relating to its implementation. It is known that the future success of an intervention depends not just on its effectiveness, but also on its reach into the population and the fidelity of its implementation (25). There are also known barriers to the translation of research evidence into clinical practice, some of which are independent of the nature of the intervention, such as the organizational readiness of the context in which it's being implemented, buy-in to the intended aims of the intervention (as opposed to current approaches), the level of training and skillset of staff required to use it, and the reputation of the person promoting the research evidence (26). The design of effective implementation strategies, for example to promote reach and overcome barriers, is a core focus of the field of Implementation Science, and such strategies might target a range of stakeholders, including service users (27) and service providers (28). Whilst there is evidence for the success of both single-component and multi-component implementation strategies, whether strategies are effective or not might have a specific dependency on whether they address known determinants such as facilitators and barriers (29), which might be specific to both an intervention and to the health system context in which it is introduced.

Work presented in this paper has taken place as part of the Narrative Experiences Online (NEON) study, a 5-year programme of work funded through the Programme Grants for Applied Research scheme of the National Institute of Health Research from 2017 until 2022, in England. The aim of NEON is to investigate whether accessing RRNs delivered online can improve quality of life for people affected by mental health problems. NEON has developed an online intervention in the form of a recommender system that matches recovery narratives to people with experience of mental health problems (30). NEON has adopted a two-armed approach to preparing for the implementation of this intervention in future healthcare practice. Arm 1 is a randomized controlled trial of the NEON Intervention with people with experience of psychosis, described at http://www.isrctn.com/ISRCTN11152837, and designed to establish effectiveness and cost-effectiveness. Arm 1 was considered an essential prerequisite for implementation, given the centrality of evidence-based medicine to global mental health practice. Arm 2 is a series of research activities to develop an implementation plan to be enacted if effectiveness and cost-effectiveness is established, incorporating contributions from a broad range of stakeholders. Given a growing interest in interventions using recorded recovery narratives, these activities will provide evidence relevant to the implementation of other interventions drawing on recorded recovery narratives more generally.

The aim of this paper is to provide foundational knowledge for the design of an effective implementation plan, for example for the NEON Intervention or other RRN-based interventions such as anti-stigma campaigns (31), by identifying possible determinants of uptake. This includes influencing the design of the NEON intervention itself, using insight gained through this early work. The objectives of the paper are to identify opportunities, barriers and enablers to the uptake of such interventions, which can then be used to refine its design and its intended implementation and deployment as appropriate. The target domains are clinical practice and clinical training, the latter included as an under-researched but potentially influential usage of RRNs within health services.

## Methods

### Study Design

Three phases of focus groups with a purposive sample of mental health workers with knowledge of the UK National Health Service (NHS) were conducted. Each focus group had 4–10 participants, selected for maximum variation on NHS profession (including non-clinical professions such as health care assistants and peer support workers) and seniority. Focus groups are an established mechanism for identifying opportunities, facilitators and barriers (32, 33). Our intention was to assemble a broad representation of experience from a wide range of roles who might have contact with recorded recovery narratives (34).

### Participants

All participants were current or recently practicing (within last 2 years) mental health workers of any discipline with an interest/experience (whether positive or negative) in using RRNs. Phase 3 participants also had interest/experience in clinical education.

### Setting

A mental health trust (provider organization) in England and teaching departments at an associated University.

### Procedures

The epistemological stance underpinning this qualitative study was critical realism, which is reflective of the belief in multiple realities, rather than a single truth, each of which is socially influenced and context-dependent (35). The analytic method of thematic analysis (36) was adopted, which is a generic, theoretically-unaligned approach that is compatible with a critical realist ontology.

The design of all topic guides was informed by consultation with a group of 20 peer support workers, who advised on the focus and content of questions. The topic guide structure was theoretically informed by Normalization Process Theory (NPT) (37). NPT is an implementation theory to understand how new practices and procedures can be embedded and normalized in routine practice within organizational settings. NPT is organized around four constructs: coherence (making sense of the work), cognitive participation (who needs to be involved), collective action (what needs to be done), and reflexive monitoring (how to evaluate and monitor). To ensure relevant implementation areas were addressed, questions in the topic guide were matched to NPT concepts: general understanding of RRNs (coherence), positioning of RRN usage within services (collective action), who uses RRNs (cognitive participation) and benefits and harms from using RRNs (reflexive monitoring).

Phase 1 investigated current and potential uses by clinicians of RRNs. A 3 min video RRN was shown to prompt a semi-structured discussion about clinician experiences and perspectives on the use of RRNs in routine settings and in clinical education. Findings from phase 1 informed the topic guides for subsequent phases.

Phase 2 explored the use of RRNs in routine clinical practice. It used a working prototype of NEON Intervention as a technology probe (38), so as to facilitate a grounded discussion about this approach. A 5 min demonstration of the NEON intervention was presented to participants. This was followed by a discussion on the use of RRNs in routine practice, which also collected specific feedback on the NEON Intervention.

Phase 3 investigated the use of RRNs in pre-registration training in fields such as nursing and psychology. Participants were shown and then discussed a demonstration of a low-fidelity prototype of a staff education module which linked to publicly-available RRNs.

Focus groups were facilitated by two researchers (JRo and either FN or CY) and conducted between March and June 2019. Each lasted between 45 and 60 min to work around clinical time commitments. All participants gave informed consent, and focus groups were recorded, transcribed and pseudonymized immediately after completion. Field notes were written by a researcher during each focus group.

### Analysis

All transcripts were coded in NVivo 12. Transcripts fragments were initially analyzed deductively in line with the pre-defined research questions, by partitioning them into two broad categories: (a) Opportunities in clinical practice and clinical education and (b) Barriers and Enablers to the use of RRNs. Alongside this, an inductive analysis was carried out to allow for content that did not fit within the deductive framework to be coded and considered. Data were analyzed thematically. One researcher (JRo) coded all the data. Two researchers (JRe and SB) independently coded two transcripts (25%) for cross-checking, with discussion between analysts on any disagreements and to refine the coding approach. Themes and sub-themes (nodes) were identified within and across these broader categories, and the structure of the categories was refined in light of inductive data, with a particular focus on constant comparison of new or discordant themes to inform refinement of categories. JRo iteratively developed themes and sub-themes with input from JRe and SB before a final framework was developed. This was then further discussed and refined in the wider research team (*n* = 7). For each sub-theme, a narrative summary was produced for presentation in this paper.

## Results

A total of 48 people took part. Participant characteristics are shown in Table 1.

**Table 1:** Participant Characteristics (*n* = 48).

**Characteristic**	***n* (%)**
**Gender**	
Male	16 (33.33)
Female	31 (64.58)
Gender Queer	1 (2.08)
**Professional discipline**	
Nurse	13 (27.08)
Psychiatrist	8 (16.67)
Occupational Therapist	4 (8.33)
Support Worker	4 (8.33)
Healthcare Assistant	4 (8.33)
Psychologist	4 (8.33)
Psychotherapist	3 (6.25)
Peer Support Worker	3 (6.25)
Other	5 (10.42)
**Length of service**	
1–5 Years	10 (20.83)
5–10 Years	7 (14.58)
10+ Years	31 (64.58)

Three themes emerged from the analysis of the data relating to opportunities for use of RRNs (rationale for clinical use, clinical considerations and rationale for educational use). Four themes emerged relating to barriers and enablers for use of RRNs (intervention design, risk, trust in online resource, information technology). Emergent themes and subthemes about the use of RRNs are shown in Table 2.

**Table 2:** Themes and sub-themes relating to opportunities, barriers and enablers for use of recorded recovery narratives.

**1. Opportunities**	
1.1 Rationale for clinical use	1.1.1 Reinforce clinical work 1.1.1.1 Reduce communication barriers 1.1.1.2 Normalization of experiences 1.1.2 Extend clinical work 1.1.2.1. Increase hope and connection 1.1.2.2. Address stigma
1.2 Clinical considerations	1.2.1 Relationship with care pathway 1.2.2 Choice of staff 1.2.3 Stage of recovery
1.3 Rationale for educational use	1.3.1 Accessing lived experience perspectives 1.3.2 Training non-clinical staff 1.3.3 Attitudinal change in trainees
**2. Barriers and enablers**	
2.1 Intervention Design	2.1.1 Ability to use online resources 2.1.2 Accessibility of language 2.1.3 Ability to individualize choice of narrative
2.2 Risk	2.2.1 Content that triggers distress 2.2.2 Staff skills to respond to negative effects
2.3 Trust in online resource	2.3.1 Evidence base 2.3.2 Maintenance
2.4 Information Technology	2.4.1 Cost of use 2.4.2 Technology requirements for use

### Opportunities

#### Rationale for Clinical Use

Participants described potential benefits of RRNs for use within clinical practice. Firstly, RRNs could enhance existing clinical work. Participants identified the use of RRNs as having potential for use as an “opener” or starting point; a way of progressing with an individual if stuck; to provide or complement information sharing; and as a resource to signpost patients to. Using RRNs as a potential tool in these instances could allow clinicians to improve engagement with patients, and support targets or plans:

“I think something like that could be a really good starting point because … some people have never even had these conversations 'would you like a job, or would you, what do you think about college' and you know it is completely new to them.” (Phase 1 Focus Group 2 (1.2): Female, occupational therapist)

RRNs might reduce the communication gap between clinician and patient:

“They want to hear it from someone else other than a professional…They want to hear it from someone with lived experience who has gone through it.” (2.2: Male, occupational therapist)

RRNs are a resource to enhance clinicians' abilities to communicate the nature of treatment option much more effectively and increase adherence:

“The number of times when I have seen a patient [and I'm] trying to explain what CBT is about, what trauma therapy is about and I think, 'I wish I had this person who can tell them what this is about', because you break down the barriers… it makes it an equal thing.” (1.3: Male, psychotherapist)

RRNs offer a way of normalizing an individual's experiences:

“Any normalization which again… some of these stories could help, to get them back out again as quickly as possible before they did become too enveloped in the mental health services.” (1:2: female, occupational therapist)

RRNs can offer clinicians a way of extending their clinical work, in particular as an option to use when “stuck” or when unable to make progress:

“If you have got the chance to [access narratives] and you don't know what else to look at because they are so, like I say, you are stuck and don't know what to do, you just think today we are going to listen to this together and then we are going to discuss what… you think of it.” (1.4: Female, psychiatrist)

More specifically, RRNs present an alternative application in the form of presenting information and testimonials from others' experiences:

“If your story was about that particular activity or that particular intervention that could be really great because you know I have struggled in the past with people with doing…or taking that first step.” (1.3: Male, psychotherapist)

RRNs were also seen to present a trusted resource to signpost patients toward:

“I think actually because people are going to try and access something, particularly the younger patients…because it isn't regulated, there are just going to be horrific places and I think if you know that that's being regulated, then therefore you'd feel safer about directing someone in that.” (2.1: Female, peer support worker)

Benefits of the potential for RRNs to increase hope and connection were identified, especially for younger patients. Hope and connection were considered to be interlinked, and the potential for a patient to develop connection with the narrative's narrator and subsequently feel inspired or hopeful for their own recovery journey was an appealing prospect:

“It is the young people with new psychosis that want hope. That is what they want, they want to know that things could potentially get better.” (1.4: Male, psychiatrist)

“I think the big one is the connectiveness - people understanding that a lot of people have similar symptoms to them.” (1.4: Male, psychiatrist)

Some participants saw a potential benefit of RRNs even if there may be a lack of connection:

“One of the things that the narrative is good for is [things] that you don't tend to get in a textbook…if you have got someone telling you their story directly in their language… it is a much more realistic way of getting to the heart of that story.” (3.1: Male, nurse)

Participants frequently referred to the use of RRNs as a way of reducing internalized stigma. In doing so, it was envisaged that a patient might remain optimistic about their own recovery journey:

“[The narrative] reduces the stigma, it reduces the fact that the individual is alone, it starts to increase the fact that 'oh thank goodness someone else has been here, this bloke has worked with someone else who has been here and there is a prognosis, there is a solution, there is a way to go forward'.” (1.3: Male, nurse.)

#### Clinical Considerations

Three considerations for clinical use of RRNs were identified: relationship with care pathway, choice of staff and stage of recovery. For some participants, engaging patients early with RRNs, for instance after an initial episode of psychosis, would be ideal to provide insight and validate what an individual has or is experiencing:

“If you think about early intervention… when things are starting to happen that they are picking up on, that potentially could be quite useful time.” (1.4: Male, psychiatrist.)

Inpatient settings were also described as ideal to use RRNs:

“Because we have time and the patient might stay a few months on the ward so you can actually try this, it might succeed, it might fail but there is opportunity, a window of opportunity here to try on inpatients.” (1.4: Male, psychiatrist)

Alternatively, RRNs could be used as an additional resource upon discharge from services:

“So maybe at the point of discharge maybe paperwork with some narratives in it, or maybe somebody who has gone through say early intervention, they have got the first period and it is something that we could give, like a recovery pack which might have narratives written in it, but it may also have links to websites or podcasts or whatever it may be.” (1.4: Male, psychiatrist)

Some participants considered peer support workers, support workers and nurses best placed to use RRNs due to the nature of engagement activities that these staff have with patients (one-to-one, time allowance):

“I think this is an amazing resource that could be used…for me, as a peer worker, this would allow other people within my team to be able to really connect with people and have those discussions that they might not otherwise be able to have, because they might not have had any experiences that they could really particularly relate to with that person or that individual.” (1.1: Female, peer support worker)

Participants only identified crisis teams as a possible area within mental health services where the use of RRNs may prove difficult to implement, due to priorities and time restraints that are placed upon staff:

“We work in the crisis team so although we are recovery focused, it is very much risk assessing and minimizing risk. So I think in that respect it would be very difficult to sort of include that into the package that we have to do in quite a short time with the… limited time that we really try to engage with people.” (1.4: Female, nurse)

Psychiatrists were considered less likely to use RRNs as their time is perceived as being too constrained or that they focus on other priorities:

“It is not suitable for the MDT or psychiatrist to spend some time with the patient showing them videos like this.” (1.4: Male, psychiatrist)

Some perceived RRNs to have the versatility to be used at different recovery stages:

“I think you could use it at the beginning, you could use it in the middle, you could use it at the end. It depends, everybody is different and I think you can have some people admitted that are, you know, really despairing and hopeless and isolated and you're offering a window to them.” (2.2: Female, nurse)

Others raised issues around capacity of a patient in order for RRNs to be effective:

“Would this be quite useful very early on - but then you say well if they are floridly psychotic that could be an issue, would it be more useful during the treatment sort of phase when the symptoms are more managed and people may be more receptive to picking these things up, or even after that when they are in remission of the symptoms.” (1.4: Male, psychiatrist)

#### Rationale for Educational Use

RRNs were identified as having a positive impact on the workforce in ways that enhance staff understanding and insight. Participants saw the use of RRNs within pre-registration training as an opportunity, particularly for nursing and psychology students, to view first person experiences of mental health difficulties:

“An essential part of the training I think, no clinician learns effective skills from studying a text book do they, it is about listening to the lived experience and all the individual nuances with that.” (1.1: Male, nurse)

For clinicians, RRNs provide a chance for trainees to see how people communicate experiences in their own words, i.e. offering a common language:

“It might give them a chance to think about how they would engage with somebody in that situation and how actually if someone is using their language, how do you in a professional way reflect that back and don't talk a lot of jargon to them or don't rephrase things in that way, how can you use shared language or a common language with somebody.” (3.1: Female, nurse)

Participants suggested that non-clinical staff such as care assistants may benefit from viewing RRNs, to gain valuable insight into patients' experiences:

“I think there is a huge potential as a means of first access for people who have had no training whatsoever and I think these are people who are neglected, these are people who misunderstand and these are ultimately people who if they are not managed properly, can create little groups of brutality you know…if you look at the appalling things that are coming to light over recent years it is often groups of care assistants who are not managed who have no understanding of the people they are looking after.” (3.1: Male, nurse)

Participants also identified RRNs as a way to increase staff empathy and compassion for clients:

“Talking about reducing the distance…is something I find particularly kind of interesting and I think narrative is really powerful doing that, both in terms of helping people feel compassion and relating to the person that is telling that, but perhaps also trying to break down some of the professional boundaries.” (3.1: Female, nurse)

RRNs may reduce or challenge any prejudices or pre-existing stereotypical attitudes some clinicians may hold:

“I think sometimes people are pre-judged so initially perhaps when you say like is this person going to tell their story, the longer the story goes on, the more you get drawn into it and you see them more as an individual rather than any kind of prejudice that you started out with, that is a very kind of powerful way of challenging stuff.” (1.1: Male, nurse.)

### Barriers and Enablers

#### Intervention Design

Particular design aspects around the use of an intervention that presents RRNs was a key theme for participants. Sub-themes were discernible, such as a recipient's ability to use and understand the intervention and having the option to tailor and provide choice to individuals.

Participants felt that many patients would struggle to use online collections of RRNs, and a lack of computer literacy may further limit access:

“It is just ensuring that it is not alienating people that don't have access, either don't have the computer skills and don't have the means.” (1.2: Female, nurse)

Language and terminology used to host online collections of RRNs pose potential limitations:

“I think it has got to be simple, it's got to be simple language.” (2.2: Female, nurse)

Participants frequently referred to the ability to tailor options within online interventions as being important and helpful for patients. Being able to choose the modality of the RRNs was thought to be a strong benefit, and was appealing from the perspective of clinicians:

“I like the way that sort of having recorded stories can be used in many different ways for instance…a video can be paused at any time, somebody can take 5 min or then if somebody is sat with them watching that video… they can have that space then to explore and discuss what that meant for them.” (2.2: Female, peer support worker.)

#### Risk

Participants identified risk and the potential to cause harm as concerns with using RRNs. The possibility that RRNs could affect a patient harmfully was a concern, particularly around issues such as self-harm:

“For some people watching videos about self-harming could be potentially triggering so there is a slight caveat, we need to be cautious about that. Equally people who have been through very traumatic experiences there is a potential to be re-traumatized by the content.” (2.2: Male, occupational therapist)

For some participants it was the unknown elements of RRNs that concerned them:

“You know it affects them, which we want it to affect them, but maybe they take it in a way that we don't think it could.” (2.2: Male, support worker)

A further risk-related area that was important for participants when using RRNs was feeling confident that they could respond to any potential effects that viewing RRNs could have on patients.

“There is probably a sense amongst professionals that they might want to be equipped with certain sets of skills before using recovery narratives - say how do you recognize cues and triggers, how do you work with people around relapse signatures?” (1.1: Male, nurse)

This was particularly notable for the capacity of clinicians to follow-up or support patients:

“So perhaps if they are using recovery stories, it is important to have someone with the person to support them through that?” (1.2: female, nurse.)

#### Trust in Online Resource

Participants discussed the potential for an online collection to act as a resource that could be trusted. A strong evidence base was vital for participants to adopt such a resource:

“Unless it is very significant and the evidence is so solid, and concrete, you can't actually do anything about it unless you have a strong evidence based stuff.” (1.4: Male, psychiatrist)

Ongoing maintenance was seen as important if an online resource was to be adopted for participants to use and signpost individuals to:

“Updating because the most irritating thing is saying oh have a look at this site and it's like well actually I phoned that number, that number doesn't exist anymore.” (2.2: Female, counseling supervisor.)

#### Information Technology

Resources within health services were identified as a major barrier for participants to make use of RRNs. This included costs related to the use of a RRNs collection, and technology required to access and make use of such a collection.

There was a strong belief that a resource should be open access and free to all:

“For me it would be important that it is open access but I would like to have that option to do both to incorporate it into the treatment plan as well.” (2.2: Male, psychotherapist)

Technology issues associated with an online collection of RRNs posed a potential barrier, for example whether patients would have the means to access such a resource outside of NHS services:

“A lot of our clients don't have IT access. We all tend to think that because the majority of us have mobile phones, a lot of our clients haven't got mobile phones, haven't got computers, haven't got internet.” (2.2: Female, nurse)

The capability of NHS technology to utilize such a resource was also questioned:

“Crap NHS computers… I question whether it could play a video.” (1.2: Female, occupational therapist.)

## Discussion

This study identified the perceptions of a range of mental health workers about opportunities, barriers and enablers of integrating RRNs into clinical practice and education. Practice opportunities include using RRNs as an approach to engagement with patients, improving hope and reducing stigma. As a method of progressing engagements with patients, their use resonates with motivational interviewing techniques particularly around reframing resistance in order to inspire and create impetus for change (39). Effective engagement within services leads to improved outcomes, particularly as a result of enhanced communication (40) and having trust in clinicians (41). RRNs may also improve connectedness and hope (42–44) which are both important for recovery (45). RRNs can also supplement peer support work, which whilst having a very strong evidence base (46–48) has human resource limitations. The opportunities afforded by RRNs are many and varied. RRNs complement existing resources in terms of their flexibility i.e., they can be deployed in a number of situations, both to complement and reinforce therapeutic input, as well as help to shift dynamics and move forward a process that might have become stuck.

Perceived barriers and enablers include the demonstration of a trusted evidence-base and sufficient upskilling of staff. Some of these influences may reflect assumptions which are not consistent with the available evidence. For example, the concern that patients lack computer literacy to engage with online resources is not supported by available empirical evidence, which suggests that patients have similar internet access and technology skills to non-patients. For example, patients' engagement behavior with the internet mirror those of non-patients (49, 50). The use of online mental health interventions, particularly in psychosis, is more feasible and acceptable in psychosis treatment than clinicians often believe (51–53). Technological aspects may of course need to be considered, such as patient concentration levels and concerns over information overload (49). Therefore, whilst areas may be identified as potential “barriers” by a particular group (in this case mental health workers), with reference to how *another* group might respond (in this case service users), care should be taken to interrogate whether there is evidence to the contrary, or whether a contrasting viewpoint might be expressed by service users. The NEON Trial has sought input from service users (30) to ensure that their own perceptions of barriers, opportunities and facilitators are also sought and considered alongside input from mental health workers.

Factors identified as barriers and enablers within this paper mainly reflect concerns of staff, as the focus groups were wholly comprised of staff members. This is why the needs of clinicians feature so heavily in the data, e.g., how narratives can help staff move forward with patients when “stuck,” and how elements associated with risk need to be addressed. The findings must be viewed as reflecting this perspective, and not necessarily reflecting needs that might be identified by service users, as mentioned above, who may identify different opportunities, barriers and enablers of RRNs. However, a significant strength of accessing mental health workers' views in particular is that from the viewpoint of NPT, if end users can identify how the introduction of a new intervention or method might help solve problems they currently face, and therefore create buy-in from staff, then it is more likely to be implemented within such services (54). The applicability of an intervention to a staff's context of work aligns with two constructs within NPT, “coherence” and “cognitive participation” (55). In addition, if staff are involved in early discussions about its use, they are able to identify reasons to support implementation in the context of its use, and highlight concerns that need to be addressed. These have been highlighted as important factors in designing complex interventions that are implementable (54), and the focus group findings have been used to influence the design of the NEON intervention. It is important to acknowledge that this approach at once helps to solve important issues that might affect its implementation, yet its potential limitations are also understood.

In this study, participants identified potential areas in clinical services that would suit the use of RRNs (e.g., during clinical appointments), areas where they might not work (e.g., in crisis situations), and challenges that may need to be overcome. These all provide a useful basis for refining the planned design and use of the RRNs in clinical settings. Themes around opportunities offered by RRNs within clinical education were concerned with providing trainees with advanced communication skills using RRNs prior to either placement or clinical opportunities began. These results support evidence suggesting that exposure to recovery narratives in professional training programmes for professionals enhances communication skills and empathy (23).

The “physician's bias/clinician's illusion” (56) refers to a when some clinicians have overly pessimistic expectations about course and outcome in psychosis, and that this can have negative consequences for organizational culture (e.g., defensive risk-orientated practice rather than positive risk-taking) and therapeutic pessimism. Stigmatizing beliefs still exist among health professionals (57). Participants believed that exposure to RRNs as part of clinical education could contribute toward attitudinal changes toward patients. Our results are consistent with the empirical evidence that suggests direct social contact involving recovery narratives is an effective evidence-based anti-stigma intervention for the community (21).

### Strengths and Limitations

Strengths of this study lie in the wide range of mental health professionals recruited, although additional participants from third sector services would have provided a broader range. The range of professionals for the clinical education focus groups were limited to trainers of nursing and clinical psychology.

We identify several limitations. First, the topic guide explored RRNs as a resource for clinical use, in line with the aims of the study. However, this de-emphasizes the important role of stories as a form of resistance, for example in expressing negative experiences about the mental health system or in expressing non-clinical understanding of experiences. The role of first-person knowledge, as expressed in RRNs, is a focus in the emerging fields of survivor research (58) and Mad Studies (59). These new perspectives support re-appraisal of existing psychiatric concepts such as insight (60). To maximize participant engagement, in this study we did not explore participant views about the use of more diverse RRNs, such as those which contain knowledge which is new to professionals, or are critical of clinical practice. As well as supporting clinical practice, RRNs are also a resource to inform professional knowledge and practice, so an important future research focus will be investigating how receiving RRNs can impact on staff, both in terms of benefits and harms.

A second limitation relates to the contested nature of recovery discussed earlier. Framing RRNs as a potential intervention or resource for use in the mental health system, as in this study, may be criticized for co-opting the narratives of individuals. Approaches which the wider NEON study is taking to address this concern include careful consideration of ethical issues to ensure our use of RRNs is consistent with the narrator's consent (61), active engagement with individuals and groups from marginalized communities specifically including people who either do not use services or have problematic relationships with services (62) to invite them to donate their narrative [www.researchintorecovery.com/donateastory], decision-making about inclusion of RRNs being made by a group whose majority membership is people with lived experience to reduce the likelihood of only pro-system RRNs being included (30), and characterization of narratives using a standardized instrument (63) which captures the full range of RRNs, including those in which the narrator explicitly rejects the concept of recovery, which allows targeted approaches to improve the diversity in the RRN collection.

Third, because the aim of this study was to collect preliminary information about using RRNs in a range of clinical and educational contexts, data collection did not continue until theoretical saturation was complete. It is therefore possible that some emergent categories would be further refined with more data collection. Fourth, the analysis could be more rigorous, for example involving more analysts second-coding more transcripts.

The final limitation relates to the focus in this study only on the views of self-selecting mental health workers. A practical implication is that the language and concepts captured in the coding framework may reflect both a positive perspective, if those who took part have more favorable views about RRNs than typical clinicians, and a focus on clinical priorities and views of the world. In other research we have focused on the impact on mental health services users of RRNs in general (12, 13, 44), but not yet on the impact of RRNs specifically when used in a clinical context. A service user perspective on clinical use of RRNs might involve different language and concepts.

### Future Research

Since using RRNs with patients could be especially relevant for peer support workers (PSWs) in their role, further work could take place to evaluate PSW views on using RRNs and inform how they are used by clinicians. It is known that experienced PSWs disclose those parts of their narrative which are most relevant to the patient they are working with (64), so capturing their expert knowledge about tailoring narratives to patient needs may inform clinical use of RRNs.

Future research may also investigate how to manage the optimal balance between receiving challenging but ultimately helpful narratives with avoiding causing unnecessary and unhelpful distress through triggering. Any evaluation of the use of narratives should include consideration of both benefits and harms (65). This involves careful consideration of available evidence, for example the currently un-resolved issue of whether content or trigger warnings are helpful (66, 67) or harmful (68).

The role of RRNs in professional education is an under-researched area. Empirical evidence about the impact of RRNs on patients is emerging (12, 13, 44), but the impact of narratives on staff, for example in increasing therapeutic optimism or reducing negative attitudes toward people with mental health problems, is an important knowledge gap. A related question is which types of RRN content are most helpful for use in professional education, and new approaches to characterizing narratives (63) mean this question can now be investigated.

The study approach and results move away from attempts to initiate a top-down approach to change in which solutions to issues are more likely to become unsuitable and fail. This study demonstrates that there are many opportunities to use RRNs, which can both reinforce and extend clinical practice and education.

## Conclusion

Overall, the findings support an increased use of RRNs in mental health education and practice. RRNs in clinical practice may be used to reinforce or extend clinical practice, and relevant clinical considerations include system structures and both staff and service user characteristics. Implementation influences to consider are the intervention design, potential harms, relationship with online resources and the digital infrastructure of the system. For the NEON study, this will inform how the intervention is described to clinicians (as an adjunct or extension rather than a replacement), the importance of local planning to integrate the intervention into existing care pathways, and the importance of addressing digital exclusion of some service users. The integration of RRNs into appropriate points on the care pathway, and the availability of accessible resources and the technological resources to access them, need to be future implementation priorities in order to “generate evidence and promote the appropriate integration and use of technologies” (p. ix) (69).

## Data Availability Statement

The datasets presented in this article are not readily available because Ethics approvals do not allow for the publishing of full transcripts. Anonymous transcripts may be available from the study sponsor on reasonable request (randienquiries@nottingham.ac.uk). Requests to access the datasets should be directed to randienquiries@nottingham.ac.uk.

## Ethics Statement

The studies involving human participants were reviewed and approved by London-West London REC and GTAC. The patients/participants provided their written informed consent to participate in this study.

## Author Contributions

JRo contributed to the design of the study, recruited participants, conducted focus groups, conducted the formal data analysis, contributed to the interpretation of the results, and wrote the first draft of the manuscript. SB and JRe conducted the formal data analysis, contributed to the interpretation of the results, and provided comments on the manuscript. SR-E, KP, and MS contributed to the design of the study, contributed to the interpretation of the results, and provided comments on the manuscript. FN and CY facilitated focus groups, contributed to the interpretation of the results and provided comments on the manuscript. JL-B, AH, PC, GT, and DM contributed to the interpretation of the results and provided comments on the manuscript. All authors read and approved the final version of the manuscript.

## Conflict of Interest

The authors declare that the research was conducted in the absence of any commercial or financial relationships that could be construed as a potential conflict of interest.
